# *Bacillus subtilis* strain UD1022 as a biocontrol agent against *Magnaporthe oryzae*, the rice blast pathogen

**DOI:** 10.1128/spectrum.00797-25

**Published:** 2025-09-22

**Authors:** Timothy Johnson, Lainey Kemmerer, Nalleli Garcia, Jessie Fernandez

**Affiliations:** 1 Department of Microbiology and Cell Science, University of Florida 3463, Gainesville, Florida, USA; Universita degli Studi del Molise, Campobasso, Italy

**Keywords:** *Bacillus subtilis *UD1022, *Magnaporthe oryzae*, rice blast, biocontrol, volatile compounds, systemic resistance, fungal inhibition

## Abstract

Rice blast disease, caused by *Magnaporthe oryzae*, is a major threat to global rice production, necessitating sustainable disease management strategies. Compared to chemical pesticides, biocontrol agents, such as beneficial microbe antagonists, offer a sustainable approach to naturally inhibit plant pathogens. This study evaluates the biocontrol potential of *Bacillus subtilis* UD1022 against *M. oryzae* through both direct antagonism and volatile-mediated inhibition. In dual culture assays, UD1022 significantly inhibited fungal growth. Furthermore, a stacking plate assay demonstrated that UD1022 produces volatile organic compounds (VOCs) that suppress fungal growth. Beyond vegetative growth inhibition, UD1022 also disrupted key infection processes of *M. oryzae*, notably reducing spore germination and appressorium formation. *In planta* assays revealed that UD1022-treated rice plants exhibited a substantial reduction in disease severity compared to untreated controls. This reduction correlated with the upregulation of key defense genes in the salicylic acid, jasmonic acid, and ethylene signaling pathways, suggesting that UD1022 primes systemic resistance in rice plants. These findings establish UD1022 as a potent biocontrol agent capable of suppressing *M. oryzae* through direct antagonism, VOC-mediated inhibition, and induction of systemic resistance. This study underscores the potential of UD1022 as an eco-friendly alternative to chemical fungicides for managing rice blast disease.

**IMPORTANCE:**

*Magnaporthe oryzae* is a destructive fungal pathogen that causes rice blast disease, leading to significant yield losses and threatening global food security. Here, we investigated the biocontrol potential of *Bacillus subtilis* UD1022, a beneficial rhizobacterium known for its plant growth-promoting and antifungal properties. Our *in vitro* and *in planta* studies revealed that UD1022 suppresses *M. oryzae* through direct antagonism, VOC-mediated inhibition, and the induction of systemic resistance in rice. These findings demonstrate UD1022 as a promising candidate for microbial-based disease management and the role of beneficial bacteria in enhancing crop protection. This research contributes to the development of sustainable agricultural practices by leveraging naturally occurring microbes to improve plant resilience and disease resistance.

## INTRODUCTION

Rice (*Oryza sativa*) is one of the most vital crops for human nutrition, serving as a primary food source for over half of the world’s population. As population size increases and global demand for rice rises, sustainable and efficient agricultural practices are essential to ensure stable production. However, plant diseases pose a major challenge to rice cultivation, significantly threatening yields and food security (1). Among the most destructive is rice blast disease, caused by the fungal pathogen *Magnaporthe oryzae* (syn. *Pyricularia oryzae*). This pathogen infects all above-ground parts of the rice plant, leading to severe yield losses (2, 3). Responsible for over 30% of annual harvested rice losses, rice blast is one of the most persistent and devastating threats to global food security, causing an estimated $66 billion in crop losses each year, enough to feed 60 million people (4, 5). Under favorable conditions, such as high humidity, frequent rainfall, and optimal temperatures ranging from 25°C to 28°C for infection and lesion development, *M. oryzae* spreads rapidly. When these conditions persist, the pathogen can cause severe outbreaks, sometimes leading to total crop failure with yield losses reaching 100% (6).

*M. oryzae* infection begins when a conidium attaches to the leaf’s surface and germinates, producing a germ tube that forms an appressorium at its tip (2, 7, 8). The appressorium is a specialized pressure-generating structure that enables the fungus to penetrate host tissue by mechanically rupturing the tough leaf cuticle (9, 10). It achieves this by generating enormous turgor pressure, which is directed through a narrow penetration structure at its base (11). Appressorial adhesion to the host cell is a crucial step in this process, facilitated by mucilage production, which enhances attachment and ensures successful infection. Once inside, *M. oryzae* develops invasive hyphae (IH), which invaginate the plant membrane to form the extra-invasive hyphal membrane compartment, a defining feature of its biotrophic phase that separates fungal structures from the host cytoplasm (12, 13). Another key component of this phase is the biotrophic interfacial complex (BIC), a specialized structure positioned initially at the tip of the primary IH and later relocating subapically in bulbous IH (14, 15). The BIC is essential for the targeted secretion of effectors, which are small fungal proteins that manipulate plant immune responses to promote successful colonization (2, 10). As the infection progresses, the pathogen transitions to a necrotrophic phase, leading to the formation of necrotic lesions (16). Within these lesions, *M. oryzae* undergoes sporulation, producing new conidia that can be dispersed by wind or rain, allowing the disease cycle to continue and spread to new host plants. Managing rice blast disease is challenging due to *M. oryzae*’s genomic plasticity and wide geographic distribution, which allow it to rapidly evolve and circumvent host resistance through transposable elements and extrachromosomal circular DNAs (17–19). Its adaptability and hemibiotrophic lifestyle undermine current control strategies, rendering resistant cultivars ineffective and reducing fungicide efficacy, ultimately posing environmental and health risks (1). To sustain rice production, alternative and more sustainable disease management strategies must be integrated with existing approaches for long-term control.

In recent decades, biological control strategies have emerged as a promising alternative to synthetic pesticides and fungicides. Biological control agents (BCAs) are living organisms or their derived components that suppress pest or pathogen populations, typically through antagonistic interactions or host priming mechanisms (20, 21). Against fungal pathogens, BCAs—often bacteria or fungi—produce secondary metabolites, antibiotics, or other bioactive compounds with fungistatic or fungicidal properties (20). Depending on the target pathogen and crop system, BCAs can be applied as soil amendments, foliar sprays, or seed coatings (22).

Beyond direct antagonism, many bacterial BCAs promote induced systemic resistance (ISR) in host plants, enhancing plant immunity through cross-kingdom signaling and priming a more robust defense response (21, 23). Some BCAs also promote plant growth independently of pathogen presence, functioning as plant growth-promoting rhizobacteria (PGPRs) by improving nutrient availability, root architecture, or plant hormone production (20). Among these, many *Bacillus* spp., including certain strains of *Bacillus subtilis*, exhibit strong potential as both BCAs and PGPRs (24).

One such strain, *Bacillus subtilis* UD1022 (hereafter referred to as UD1022), originally obtained from Dr. Harsh Bais at the University of Delaware, has been commercialized for its ability to enhance plant growth and provide disease protection across various crop species (25). UD1022 is a gram-positive, exopolysaccharide-producing soil bacterium with a fully sequenced genome (26, 27). It produces small-molecule antimicrobial compounds, including the cyclo-peptide surfactin, and forms robust biofilms that contribute to its biocontrol function (25).

In addition to its PGPR benefits, UD1022 has demonstrated antagonistic activity against several phytopathogens, including *Colletotrichum trifolii*, *Ascochyta medicaginicola*, *Phytophthora medicaginis*, and *Clarireedia jacksonii* (27, 28). However, its potential to control rice pathogens, particularly *M. oryzae*, remains unexplored. Given its effectiveness against other economically significant fungal pathogens, this study investigates UD1022’s antagonistic interactions with *M. oryzae* and the mechanisms underlying its inhibitory effects. Through both *in vitro* and *in planta* assays, we demonstrate that UD1022 exhibits strong antagonistic activity against *M. oryzae*, likely mediated by a combination of direct antifungal mechanisms, including the production of volatile and non-volatile compounds, and ISR-induced plant defenses. These findings demonstrate UD1022’s potential as an effective biocontrol agent in the rice blast pathosystem and its role in integrated disease management strategies for sustainable rice production.

## RESULTS

### *In vitro* inhibition of *M. oryzae* by *B. subtilis* UD1022

**Fig 1:**
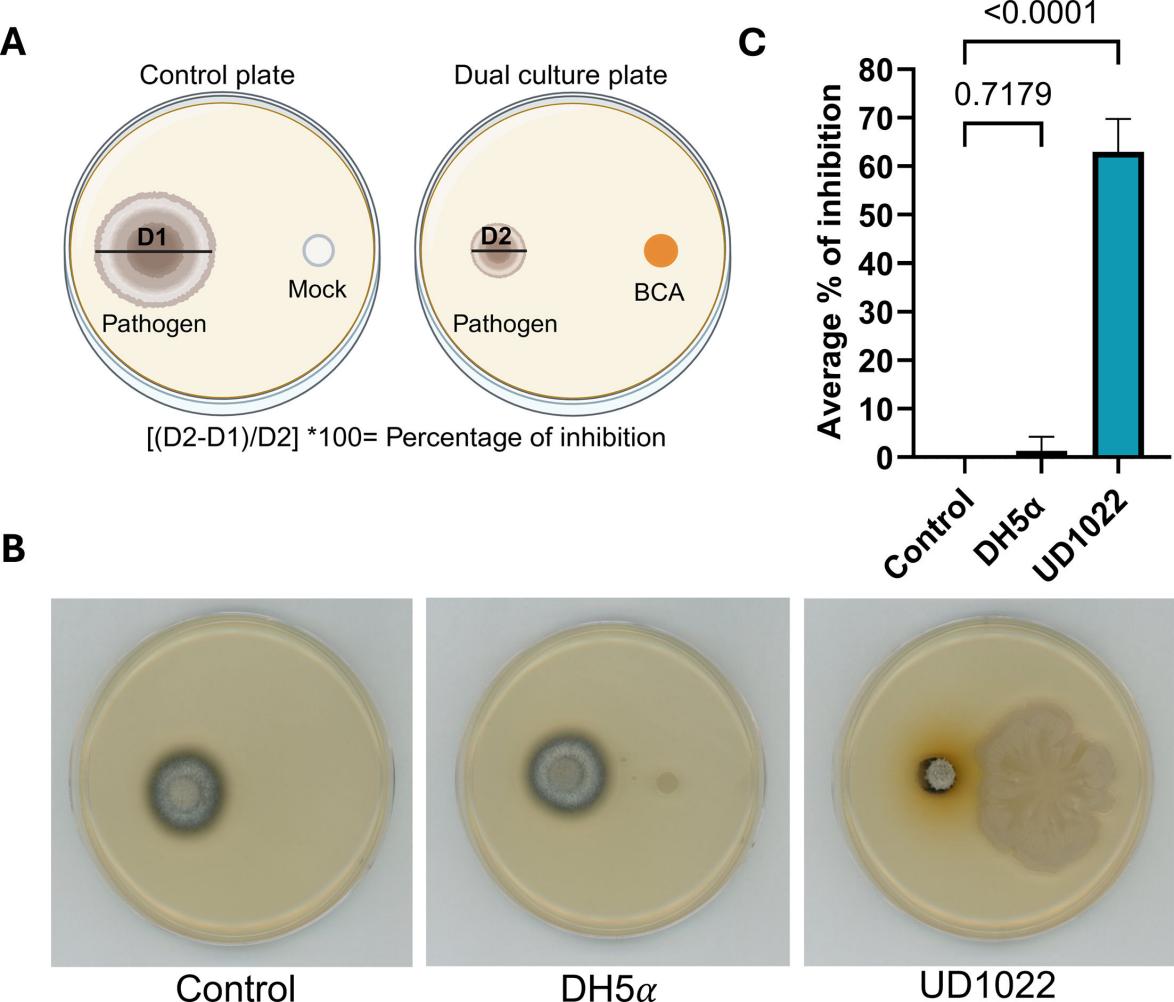
Antagonistic activity of *B. subtilis* UD1022 against *M. oryzae*. (**A**) Diagram of the dual culture assay, where the control plate contains the pathogen with a water mock, and the dual culture plate contains the pathogen with the biocontrol agent. D1/D2 represents the pathogen’s diameter. (**B**) Dual culture assay on complete medium plates. (**C**) Percentage of fungal growth inhibition in the presence of UD1022 and control samples (DH5α and water). Plates were incubated at 25°C for 5 days. Images and measurements were taken at 5 dpi. Bars represent the mean ± SD from three independent biological replicates (*n* = 3). Statistical analysis was performed using one-way analysis of variance followed by Dunnett’s multiple comparison test. Exact *P* values are indicated on the graph.

To evaluate the biocontrol potential of UD1022 against *M. oryzae*, we conducted dual culture and volatile-mediated inhibition assays using the wild-type fungal isolate, Guy11. In a dual culture assay, UD1022 significantly inhibited fungal growth by 62.9% ± 6.82% at 5 days post-incubation (dpi; *P* < 0.0001, *n* = 3), whereas the negative control *Escherichia coli* DH5α showed no inhibition (*P*  =  0.9988, *n* = 3; Fig. 1A through C). To more accurately capture the dynamics of fungal suppression over time, we also assessed inhibition at a later time point. At 10 dpi, UD1022 continued to suppress *M. oryzae* growth, with a 50.1% ± 1.67% reduction compared to the untreated controls (*P*  <  0.0001, *n* = 3), while *E. coli* DH5α-treated plates showed no significant effect (*P*  =  0.5256, *n* = 3; Fig. S1A and B).

To rule out the possibility that differential bacterial growth or media compatibility influenced the results, we assessed the growth of *E. coli* DH5α in both Luria-Bertani (LB) and complete medium (CM) at 25°C. Although *E. coli* DH5α grew more slowly in CM than in LB during the early stages (Fig. S1C), it remained viable and eventually reached comparable cell densities in liquid culture (data not shown). Unlike UD1022, which expands readily across solid media likely due to swarming motility, *E. coli* DH5α does not exhibit surface spreading. To mimic UD1022’s colony coverage, *E. coli* DH5α was manually streaked across a larger area of CM agar (DH5α-S) and incubated for up to 10 days (Fig. S1A and B). Despite this increased surface coverage, *E. coli* DH5α did not inhibit *M. oryzae* mycelial growth. These observations confirm that *E. coli* DH5α is capable of growing under assay conditions and that its lack of antifungal activity is not attributable to poor viability or limited coverage. Additionally, fungal growth on mock-treated plates remained unrestricted, indicating that the CM medium itself does not interfere with fungal development. Together, these findings highlight the specificity and robustness of UD1022’s antagonistic activity against *M. oryzae*.

**Fig 2:**
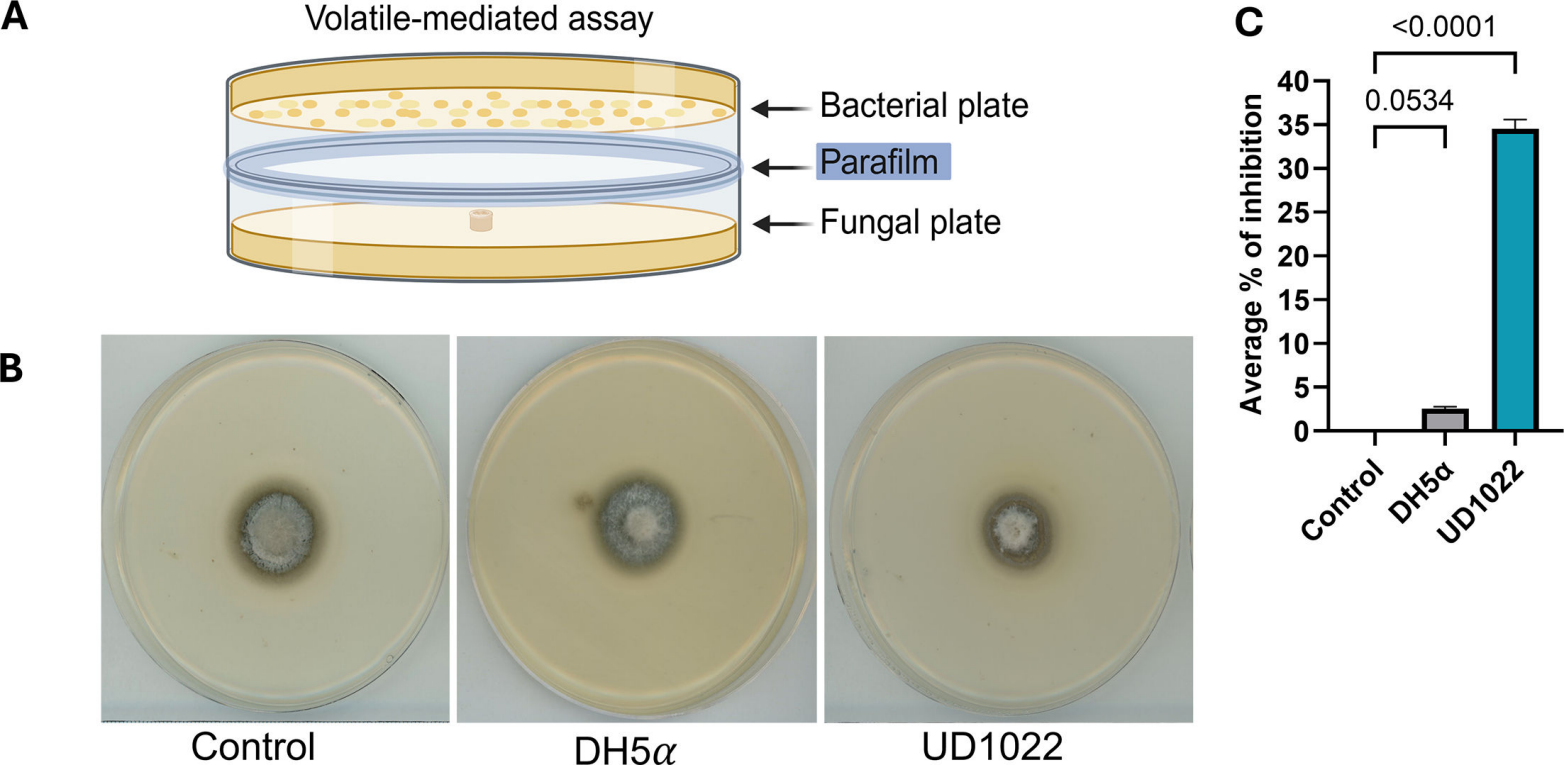
Volatility assay of *B. subtilis* UD1022 against *M. oryzae*. (**A**) Diagram illustrating the volatile-mediated assay using a stacked plate method. (**B**) Fungal plates from the volatile-mediated assay after 5 dpi. (**C**) Quantification of the percentage of fungal growth inhibition in the presence of UD1022 and control samples (DH5α and water-control). Plates were incubated at 25°C for 5 days. Images and measurements were taken at 5 dpi. Bars represent the mean ± SD from three independent biological replicates (*n* = 3). Statistical analysis was performed using one-way analysis of variance followed by Dunnett’s multiple comparison test. Exact *P* values are indicated on the graph.

Once UD1022 was confirmed to exhibit direct antagonism against *M. oryzae*, we evaluated its ability to secrete volatile organic compounds (VOCs) using the stacking plate approach (Fig. 2A). Results demonstrated that UD1022 generates VOCs capable of significantly inhibiting *M. oryzae* growth by approximately 34.5% ± 1.87% (*P* < 0.0001, *n* = 3), while the negative control *E. coli* DH5α (*P* = 0.0534, *n* = 3) and untreated control showed no *M. oryzae* growth inhibition (Fig. 2B and C). These findings demonstrate that UD1022 produces biologically active VOCs capable of suppressing *M. oryzae* growth. When combined with the results of the dual culture assay, these data highlight UD1022’s potential as a biocontrol agent that employs both contact-dependent and volatile-mediated mechanisms to inhibit fungal development.

### Inhibition of spore germination and appressorium formation

**Fig 3:**
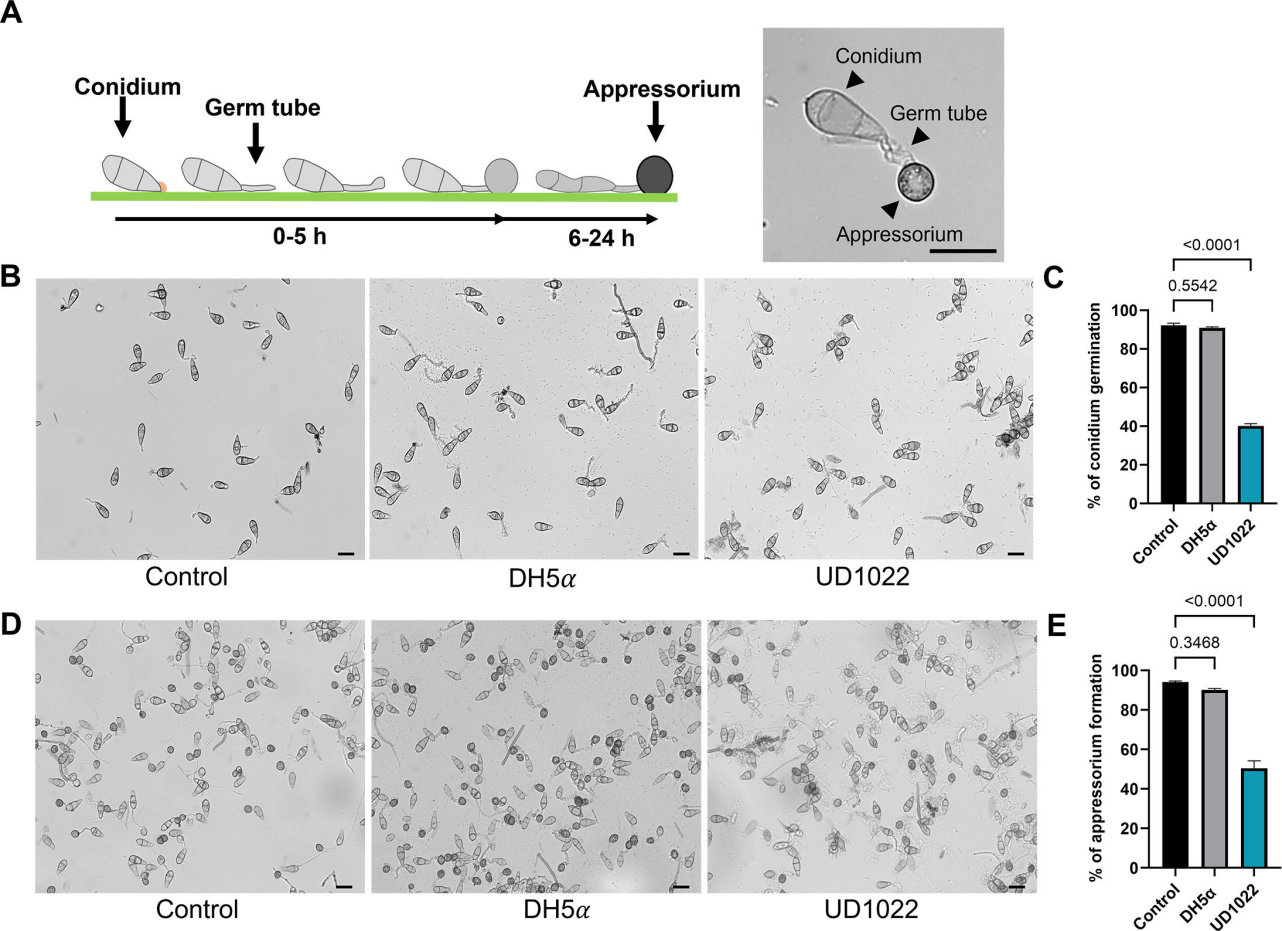
*B. subtilis* UD1022 inhibits both *M. oryzae* conidial germination and appressorium formation. Fungal conidia (1 × 10⁵ spores/mL) were mixed with bacterial suspensions (1.6 × 10⁸ cells/mL) and incubated as 20 µL drops on hydrophobic coverslips to induce conidial germination and appressorium formation. Treatments included UD1022, *E. coli* DH5α, and a sterile water-control. (**A**) Schematic representation of the stages of *M. oryzae* conidial germination and appressorium development. Representative image of a germinated conidium with a germ tube and appressorium. (**B**) Microscopy images at 3 hpi showing conidial germination under different treatments. (**C**) Quantification of conidial germination (%) at 3 hpi. (**D**) Microscopy images at 20 hpi showing appressorium formation on coverslips. (**E**) Quantification of appressorium formation (%) at 20 hpi. For each treatment, 50 conidia were counted in three technical replicates (150 conidia total per biological replicate), with three independent biological replicates (*n* = 3). Bars represent the mean ± SD. Bar graphs represent the percentage of *M. oryzae* conidial germination (**C**) and appressorium formation (**E**) under control conditions (black and gray) and in the presence of *B. subtilis* UD1022 (blue). Statistical significance was determined using one-way analysis of variance followed by Dunnett’s multiple comparison test (*P* < 0.05). Exact *P* values are shown on the graphs. Scale bar = 20 µm. The figure was created in Biorender.com.

To illustrate the initial stages of *M. oryzae* infection, we generated a schematic depicting conidial germination and appressorium development on a hydrophobic surface (Fig. 3A). Germination typically occurred within 0–3 hours post-inoculation (hpi), followed by germ tube elongation and appressorium formation between 6 and 24 hpi. The appressorium undergoes melanization, which drives its maturation and enables the buildup of turgor pressure necessary for host penetration. Figure 3A summarizes these developmental stages as visualized on hydrophobic glass coverslips, which promote the formation of infection structures *in vitro*.

To evaluate the inhibitory effects of UD1022 on the germination and appressorium formation of *M. oryzae* spores, we conducted direct interaction assays using hydrophobic glass coverslips (Fig. 3A). These surfaces mimic the hardness and hydrophobicity of plant leaves, promoting *M. oryzae* germination and differentiation (9, 29). Bacterial suspensions were mixed with fungal spores and incubated on coverslips. Conidial germination and appressorium formation were assessed at 3 and 20 hpi, respectively. Results showed that *M. oryzae* spore germination was significantly inhibited by UD1022, with only 40.0% ± 2.9% of conidia germinating (*P* < 0.0001, *n* = 3), compared to 90.8% ± 1.30% with *E. coli* DH5α and 92.2% ± 2.4% in the untreated control (Fig. 3B and C). Additionally, appressorium formation was similarly reduced to 50.48% ± 6.03% at 20 hpi on coverslips treated with UD1022 (*P* < 0.0001, *n* = 3), compared to 94.0% ± 3.03% in the untreated control (Fig. 3D and E; Fig. S2). *E. coli* DH5α shows no significant differences on both conidial germination or appressorium formation assays (90.8% ± 1.30%, *P* = 0.5542, and 91.0% ± 3.74%, *P* = 0.3468, respectively). These findings demonstrate that UD1022 significantly suppresses early infection-related development in *M. oryzae*, highlighting its promising role as a biocontrol agent.

### UD1022 primes rice root resistance against *M. oryzae*

**Fig 4:**
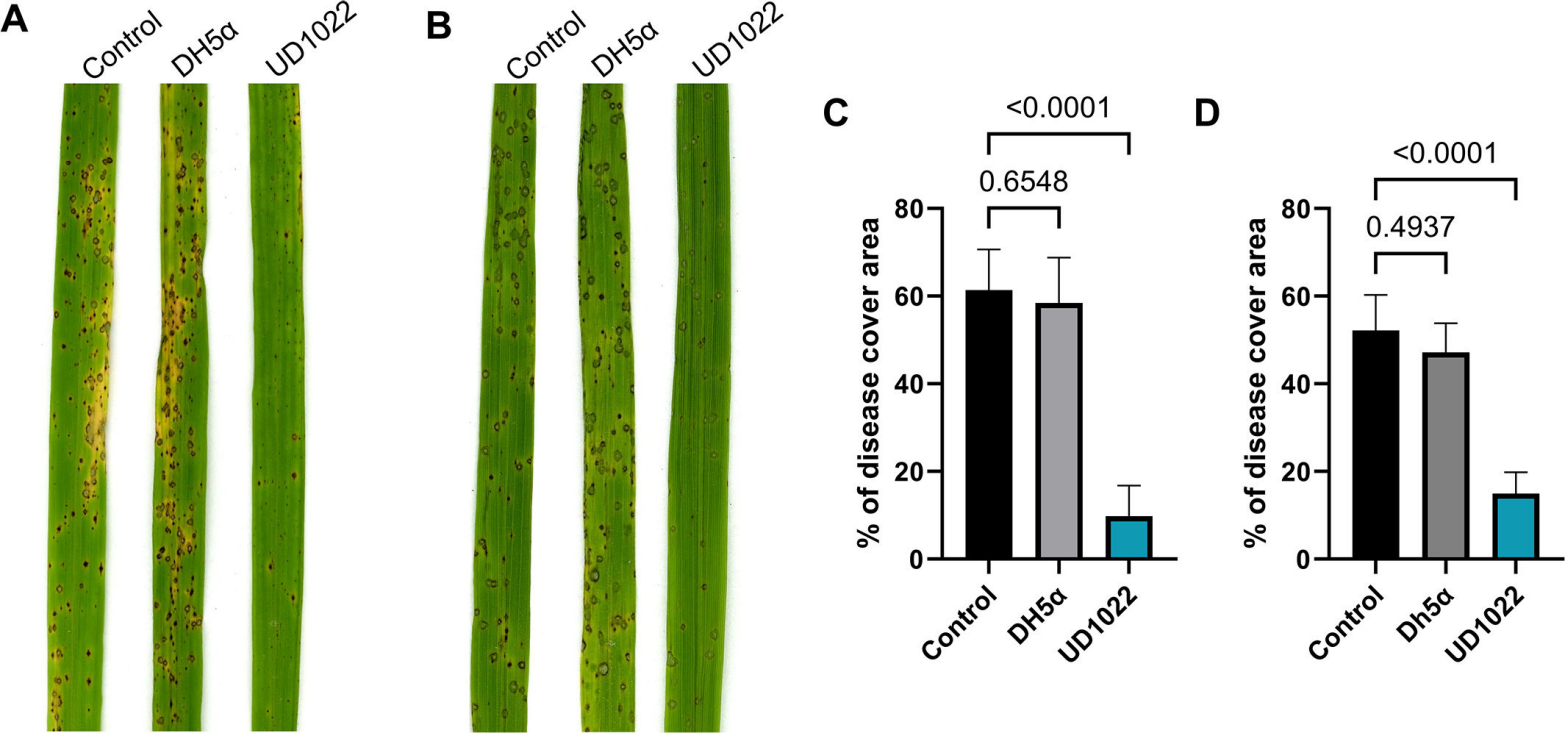
*B. subtilis* UD1022 primes rice root resistance against *M. oryzae*. (**A and B**) Representative rice leaf segments showing disease symptoms in water (control), *E. coli* DH5α, and UD1022-treated plants. Rice plants were root-primed with bacterial suspension (1 × 10^8^ cells/mL). After 24 hpi, CO39 (**A**) and YT16 (**B**) were spray-inoculated with an *M. oryzae* spore suspension (1 × 10⁵ spores/mL). (**C and D**) Quantification of disease severity, represented as the percentage of leaf area covered by lesions in CO39 (**C**) and YT16 (**D**) rice cultivars. Bar graphs show the percentage of *M. oryzae* appressorium formation under control (water-treated, black), *E. coli* DH5α-treated (gray), and *B. subtilis* UD1022-treated (blue) conditions. Error bars represent SD. Statistical significance was assessed by one-way analysis of variance with multiple comparisons (*P* < 0.05, *n* = 3).

To assess the efficacy of UD1022 in controlling *M. oryzae* infection, we conducted a bacterial inoculation followed by a pathogenicity assay to measure disease severity on rice plants. This assay was designed to determine whether UD1022 could reduce lesion development caused by *M. oryzae*. Roots of rice plants, including the moderately susceptible cultivar CO39 and the highly susceptible cultivar YT16, were treated with UD1022 suspensions and challenged with *M. oryzae* spores on the leaves after 24 hours (Fig. 4). Disease symptoms were quantified by measuring the percentage of disease-covered leaf area using ImageJ software. In CO39, UD1022-treated plants exhibited significantly reduced disease severity, with 10.24% ± 2.85% lesion coverage compared to 61.39% ± 9.25% in the untreated control (*P* < 0.0001, *n* = 3) and 58.41% ± 10.35% in the DH5α treatment (Fig. 4A and C). Similarly, in YT16, UD1022-treated plants showed 14.97% ± 4.86% lesion coverage, significantly lower than the control (52.17% ± 8.11%, *P* < 0.0001) and the DH5α treatment (47.18% ± 6.65%) (Fig. 4B and D). No significant difference was observed between the untreated control and DH5α treatments in either cultivar. Plants inoculated with UD1022 displayed significantly fewer leaf lesions compared to the untreated *M. oryzae* Guy11 control (Fig. 4A and B). While the control leaves showed extensive necrotic lesions characterized by large, irregular spots covering a significant portion of the leaf surface, UD1022-treated leaves had a markedly reduced lesion number. These findings suggest that root colonization by UD1022 activates plant-driven mechanisms that prime rice for foliar defense against *M. oryzae*, leading to an enhanced immune response and reduced infection in the aerial parts of the plant.

### Defense gene activation in rice by *B. subtilis* UD1022

**Fig 5:**
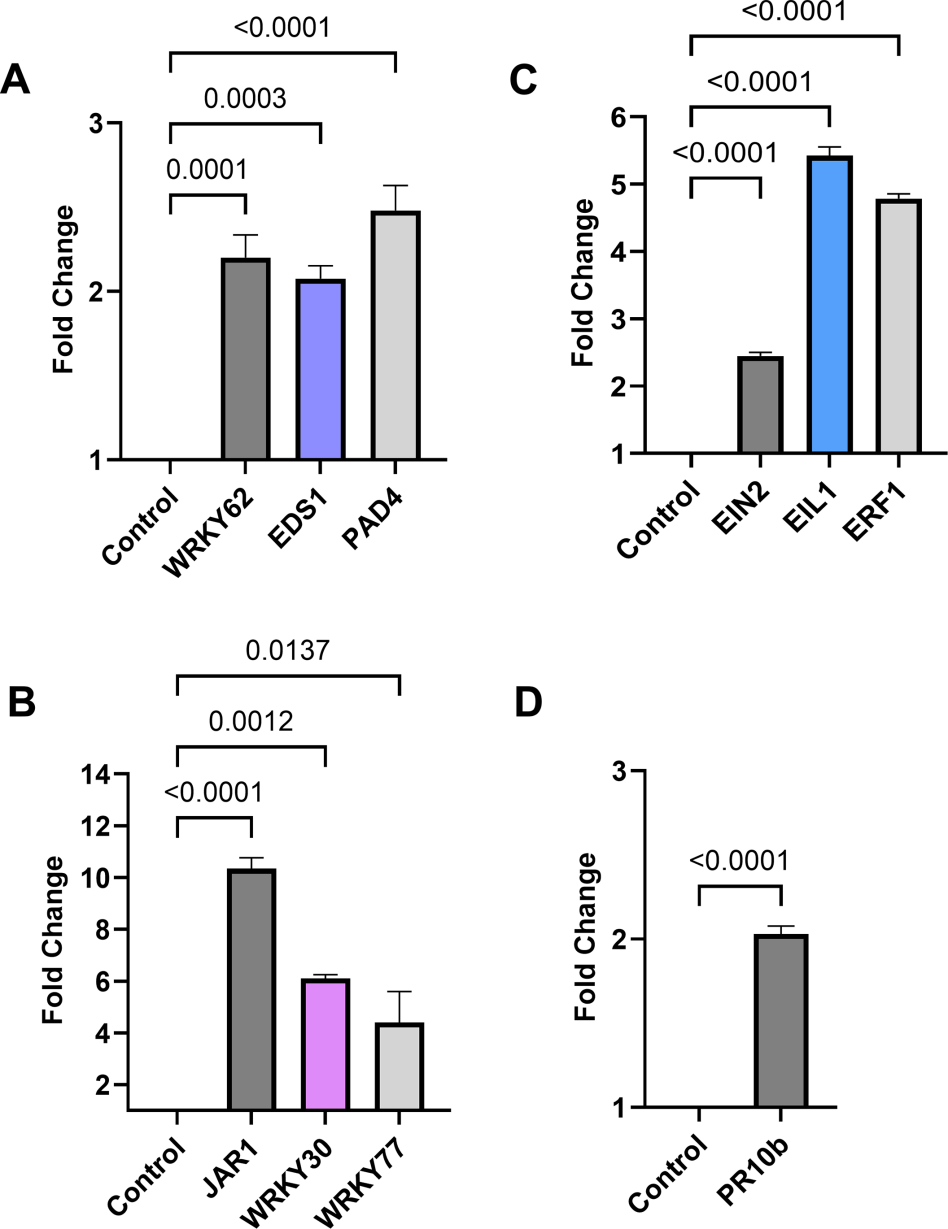
Expression of defense-related genes in rice plants treated with *B. subtilis* UD1022. Roots of aseptically grown rice plants were treated with *B. subtilis* UD1022 and water (control). Leaf samples were collected 24 hours post-treatment, and the expression of genes involved in (**A**) salicylic acid, (**B**) jasmonic acid, (**C**) ethylene signaling pathways, or (**D**) general defense response was analyzed via quantitative PCR. Samples were normalized against housekeeping genes and then to the control sample. Error bars represent SD. Statistical significance was determined using one-way analysis of variance and Student’s *t*-test (*P* < 0.05).

To further investigate the role of UD1022 in enhancing rice defense against *M. oryzae*, we examined the expression of key defense-related genes 24 hours post-bacterial treatment (Fig. 5; Table S1). We focused on the salicylic acid (SA), jasmonic acid (JA), and ethylene (ET) pathways due to their central roles in plant immunity: SA is crucial for defense against biotrophic pathogens; JA mediates responses to necrotrophic pathogens and herbivory; and ET regulates stress signaling and cross-talk between pathways (30).

We selected genes previously identified as being activated in rice and *Arabidopsis* during pathogen infection and involved in ISR triggered by beneficial PGPRs, using them as molecular markers to evaluate UD1022’s impact on hormonal defense signaling. Following UD1022 treatment, there was significant upregulation (*P* < 0.05) of SA-responsive genes *PAD4* (2.48 ± 0.26), *EDS1* (2.08 ± 0.13), and *WRKY62* (2.20 ± 0.23); JA-responsive genes *WRKY30* (6.11 ± 0.24), *JAR1* (10.34 ± 0.73), and *WRKY77* (4.41 ± 2.07); and ET-responsive genes *EIN2* (2.45 ± 0.10), *EIL1* (5.43 ± 0.22), and *ERF1* (4.79 ± 0.12), indicating activation of multiple defense pathways (Fig. 5A through C). Notably, these changes in gene expression were observed in the absence of pathogen challenge, indicating that UD1022 alone is sufficient to activate systemic defense responses in rice. The moderate upregulation of pathogenesis-related (PR) genes (e.g., *PR10b* [2.03 ± 0.07] and *PAD4*) suggests that UD1022 induces a primed immune state where the plant’s defense mechanisms are preconditioned for a faster and stronger response upon pathogen attack (Fig. 5D). Furthermore, the upregulation of WRKY transcription factors in UD1022-treated plants suggests that UD1022 modulates transcriptional reprogramming of defense networks, reinforcing its role in enhancing systemic resistance. Taken together, UD1022 root inoculation significantly upregulated defense-related genes, indicating activation of key immune pathways. This suggests that UD1022 primes systemic immunity through SA, JA, and ET signaling, enhancing the plant’s ability to resist *M. oryzae* and highlighting its potential as a sustainable biocontrol agent.

### VOCs identified in UD1022

**Fig 6:**
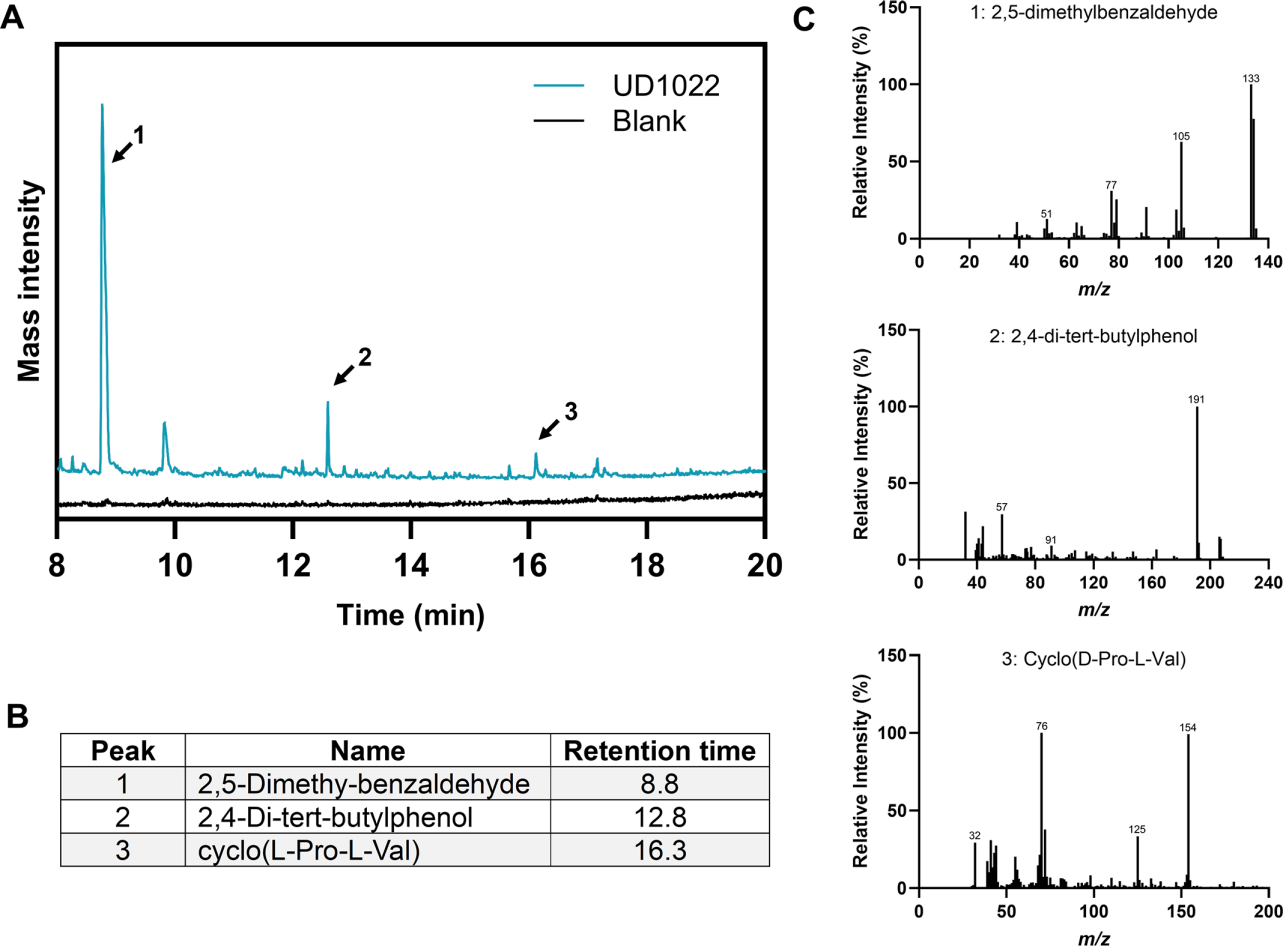
Volatile compound profile of UD1022. (**A**) GC-MS chromatogram showing VOCs produced by UD1022 after 24 hours of growth. The numbered peaks indicate the compounds identified. (**B**) Table summarizing the major identified VOCs, along with their corresponding retention times. (**C**) Mass spectra of the identified VOCs produced by UD1022, with compound identification performed using the NIST library.

To identify potential VOCs produced by *B. subtilis* UD1022 that may contribute to the inhibition of *M. oryzae*, we performed gas chromatography-mass spectrometry (GC-MS) analysis of bacterial culture headspace after allowing the VOCs to accumulate for 24 hours. Three major peaks were identified based on National Institute of Standards and Technology (NIST) library matches: 2,5-dimethylbenzaldehyde, 2,4-di-tert-butylphenol, and cyclo(D-Pro-L-Val) (Fig. 6A through C). Retention times were consistent across replicates. Mass spectral analysis confirmed the identities of these VOCs based on their characteristic fragmentation patterns and strong matches with the NIST library (Fig. 6C). Both 2,4-di-tert-butylphenol and 2,5-dimethylbenzaldehyde showed high peak intensities, indicating strong emission and volatility. Notably, 2,5-dimethylbenzaldehyde is a derivative of benzaldehyde, a well-known *Bacillus*-associated VOC (31). In contrast, cyclo(D-Pro-L-Val), a diketopiperazine (DPK) compound, was detected at lower intensity, consistent with its lower volatility. However, this compound is primarily extracted from cell-free media and is not typically classified as a volatile compound (32). Both benzaldehyde and 2,4-ditert-butylphenol have previously been reported as *Bacillus*-derived VOCs with antifungal or plant-associated activity (33–35). Their potential role in inhibiting *M. oryzae*, as observed in stacking plate assays, requires further functional testing.

## DISCUSSION

Rice blast disease, caused by the devastating pathogen *M. oryzae*, poses a significant threat to global food security by causing substantial yield losses in rice cultivation. Sustainable management strategies are needed to reduce reliance on synthetic fungicides, which present environmental risks and drive pathogen resistance. This study emphasizes the potential of *B. subtilis* UD1022 as a biocontrol agent capable of suppressing rice blast symptoms through both antagonism activity and activation of plant defense mechanisms. To our knowledge, this is the first report demonstrating UD1022’s ability to control rice blast through these dual mechanisms. In addition to inhibiting *M. oryzae* vegetative growth, UD1022 also reduces its pathogenicity by suppressing appressorium formation.

Our dual culture assays demonstrated that UD1022 significantly inhibited *M. oryzae* vegetative growth, reducing fungal colony diameter. UD1022 was previously identified as a PGPR with demonstrated antagonistic activity against oomycetes and several fungal pathogens affecting turfgrass, alfalfa, and *Arabidopsis* plants (25, 27, 28). This gram-positive bacterium readily forms biofilm and produces a diverse range of secondary metabolites, both of which contribute to its biocontrol potential. Efficient biofilm formation, coupled with surfactin production, appears to be crucial for direct antagonistic interactions (28). Previous studies link UD1022’s antifungal activity to non-ribosomal peptide (NRP) production and biofilm formation (28). Surfactin, synthesized by srfAC, partially contributed to *Ascochyta medicaginicola* inhibition, while additional sfp-dependent metabolites likely targeted *Phytophthora medicaginis* (28). However, surfactin alone failed to inhibit *P. medicaginis*, suggesting the involvement of other antimicrobial compounds. Furthermore, biofilm-deficient mutants exhibited reduced antagonism, underscoring the importance of surface colonization in pathogen suppression (28, 36). The global regulator Spo0A was found to be essential for full antagonistic activity, orchestrating NRP synthesis, biofilm formation, and motility (28). These mechanisms could similarly contribute to UD1022’s inhibition of *M. oryzae* in its vegetative form, though further research is needed.

Bacterial volatile compounds (BVCs) are low-molecular-weight compounds capable of diffusing over long distances and influencing plant growth and defense responses (37). Many *Bacillus* spp. produce BVCs with antifungal activity, though their effectiveness varies among pathogens (31). In this study, volatile compound detection was performed using a stacking plate assay, revealing that *B. subtilis* UD1022 emitted VOCs that inhibited *M. oryzae* growth on plates. This finding contrasts with previous observations in other plant-pathogen systems, where UD1022 inhibited fungal growth of *C. jacksonii*, the causative agent of dollar spot disease in turfgrass, with no effect from bacterial volatiles or cell-free filtrates, indicating that direct contact with live cells is required (27). These results suggest that the effect of UD1022-derived VOCs may be pathogen specific.

To investigate the chemical basis of this inhibition, we performed GC-MS analysis of UD1022 culture headspace after allowing VOCs to accumulate for 24 hours. Three compounds matching known bioactive volatiles were identified: 2,5-dimethylbenzaldehyde, 2,4-di-tert-butylphenol, and cyclo(D-Pro-L-Val). The first two exhibited strong peak intensities and have been reported as key antimicrobial VOCs in other *Bacillus* strains. Notably, 2,4-di-tert-butylphenol and benzaldehyde analogs were identified as dominant VOCs in *B. subtilis* CF-3, where they showed potent antifungal activity against *Monilinia fructicola* and *Colletotrichum gloeosporioides* (33, 38, 39). In that system, 2,4-di-tert-butylphenol disrupted fungal cell structures, reduced membrane fluidity and ergosterol content, and inhibited enzymes involved in host infection, such as pectinase and cellulase (33). It also induced defense-related enzymes, including POD, CAT, and β-1,3-glucanase, suggesting a dual role in both direct pathogen suppression and host immune activation. Benzaldehyde levels in CF-3 were highest at 24 hours, coinciding with peak antifungal activity, highlighting the functional importance of this compound during early-stage biocontrol (31, 39). In our study, the presence of 2,5-dimethylbenzaldehyde and 2,4-di-tert-butylphenol in UD1022 may reflect similar mechanisms of action. Cyclo(D-Pro-L-Val), although not a true VOC, has a well-documented antifungal and defense-priming profile (32, 40). It was previously isolated from *Bacillus thuringiensis* JCK-1233 and shown to be part of a class of DPKs that can induce resistance in host plants (32). In pine seedlings, DPKs, including cyclo(D-Pro-L-Val) and related compounds, suppressed pine wilt disease severity and triggered the expression of multiple PR-related genes (32). While its low volatility explains the weak GC-MS signal, its biological relevance remains high, and it may function as a semi-volatile or contact-activated signaling molecule. Additionally, further research is needed to determine the exact mechanism by which VOCs reduce *M. oryzae* mycelial growth in the stacking assay and whether this inhibition affects the formation or function of appressoria, a specialized infection structure. Since appressoria formation is fully mature by approximately 20 hpi, it is also important to investigate whether VOCs require a longer exposure time beyond 20 hpi to accumulate to inhibitory levels.

In this study, we found that UD1022 disrupted spore germination and appressorium formation—two critical stages in the *M. oryzae* infection cycle—on a surface conducive to appressorium development. The appressorium is a specialized structure essential for host penetration and colonization. By inhibiting these processes, UD1022 directly interferes with the pathogen’s ability to establish infection. Similar inhibitory effects have been observed with other *Bacillus* spp. and biocontrol agents, revealing the importance of targeting early infection stages to mitigate disease progression (41–44). Previous studies have demonstrated that *B. subtilis* disrupts cell wall integrity and inhibits appressorium formation in *M. oryzae*, effectively suppressing rice blast disease. For instance, *B. subtilis* strain G5 disrupts mycelial integrity, inhibits fungal growth, and interferes with appressorium formation, thereby reducing the pathogenicity of *M. oryzae* (41). Similarly, *Pseudomonas chlororaphis* EA105 has been shown to inhibit conidia germination and appressorium formation through direct interactions on hydrophobic surfaces (45). Further studies are needed to explore the direct effects of UD1022 on appressorium development and its underlying mechanisms.

*Bacillus* spp. are well-established biocontrol agents that enhance plant resistance against pathogens through multiple mechanisms. They produce antimicrobial compounds, such as lipopeptides, which directly inhibit pathogen growth. Beyond disease suppression, *Bacillus* spp., including UD1022, function as PGPR by regulating plant physiology and enhancing nutrient availability. They achieve this through auxin (indole-3-acetic acid) biosynthesis, siderophore production, and the solubilization of soil nutrients, all of which contribute to plant health and resilience (46). These traits make *Bacillus* spp. valuable for improving crop productivity while reducing dependence on chemical fertilizers and pesticides.

In addition to their growth-promoting properties, *Bacillus* spp. can promote ISR in plants by activating defense-related pathways. ISR is a priming mechanism that enhances the plant’s ability to respond to future pathogen attacks more rapidly and effectively. Unlike systemic acquired resistance, which is triggered by pathogen attack and leads to a prolonged immune response, ISR enhances the plant’s sensitivity to defense hormones such as JA, SA, and ET, facilitating a more rapid immune response (30, 47, 48).

The activation of ISR by beneficial microbes is initiated through recognition of microbial-associated molecular patterns (MAMPs), similar to pattern-triggered immunity, but with key differences. Beneficial microbes can either modify their MAMPs to induce a weaker and transient immune response or actively suppress immune signaling through secondary metabolites, suggesting a long evolutionary history of plant-microbe symbiosis (30). In addition to hormonal priming, ISR leads to structural, epigenetic, and transcriptomic modifications, collectively strengthening plant defenses against future pathogen challenges.

In our study, treatment with UD1022 significantly reduced rice blast symptoms while upregulating key defense-related genes, suggesting that UD1022 not only directly inhibits *M. oryzae* but also primes rice plants for enhanced resistance via ISR. Rice plants treated with UD1022 exhibited a dramatic reduction in disease severity compared to untreated controls. Treated plants developed fewer necrotic lesions in both rice cultivars tested, demonstrating UD1022’s potential as an effective biocontrol agent for rice blast.

UD1022 treatments applied to the root system effectively induced ISR, priming plant responses in the phyllosphere and enhancing resistance to pathogen invasion. Gene expression analysis revealed significant upregulation of SA-responsive genes (*WRKY62*, *EDS1*, and *PAD4*), JA-responsive genes (*JAR1*, *WRKY30*, and *WRKY77*), and ET-responsive genes (*EIL1*, *ERF1*, and *EIN2*). While PR10b has been previously identified as an SA-responsive gene, it also responds to JA signaling, indicating cross-talk between these pathways (49, 50). These findings align with previous studies demonstrating UD1022’s capacity to trigger plant defense mechanisms. For instance, previous studies have shown that UD1022 (formerly known as *B. subtilis* FB17) limits the entry of *Pseudomonas syringae* DC3000 into *A. thaliana* by triggering ISR, which induces stomatal closure in light-adapted plants, thereby reducing pathogen invasion (51). The moderate upregulation of PR genes (*PR10b* and *PAD4*) further supports the conclusion that UD1022 induces immune priming rather than full defense activation, allowing the plant to maintain metabolic efficiency while remaining in a heightened state of immune readiness. To build on these findings, future studies should assess defense gene expression after *M. oryzae* infection in UD1022-treated plants using a time-course approach, as priming responses are dynamic and may vary in both timing and intensity, depending on the infection stage. This would help capture the full spectrum of induced systemic resistance and provide deeper insight into how UD1022 enhances host defense upon pathogen challenge. Therefore, this suggests that ISR does not lead to constitutive PR gene expression but rather enhances their responsiveness to future pathogen challenge (52, 53).

Integrating multiple biocontrol agents and plant growth promoters into synthetic microbial communities offers an environmentally sustainable alternative to conventional fertilizers and pesticides. However, designing a robust community capable of withstanding diverse environmental challenges remains a significant hurdle (54). A major challenge lies in translating laboratory-based findings to field conditions, where climatic variability, nutrient fluctuations, and increased microbial diversity influence biocontrol efficacy. These factors not only affect the interaction between the BCA and its target pathogen but also shape its relationship with the host plant and native microbiome. For instance, *Bacillus* spp. have been shown to either enhance or reduce soil microbial diversity post-inoculation, suggesting potential competitive interactions that may impact beneficial microbes (55). Therefore, it is essential to evaluate both the pathogen-targeting ability of BCAs and their broader ecological impact on the host microbiome.

This study establishes *B. subtilis* UD1022 as a promising biocontrol agent for managing rice blast disease. Its dual mechanisms of direct antagonism and ISR activation provide a comprehensive approach to disease suppression, reducing reliance on chemical inputs and supporting sustainable agriculture. To our knowledge, this is the first report of UD1022 reducing rice blast symptoms. Further research should validate UD1022’s efficacy in field conditions, explore its interactions with native soil microbiota, and optimize its formulation for sustainable, large-scale application in rice blast management.

### Conclusion

In this study, we demonstrate that *B. subtilis* UD1022 effectively suppresses *M. oryzae* growth and infection through multiple mechanisms, including direct antagonism, VOC-mediated inhibition, and the induction of systemic resistance in rice plants. Dual culture and stacking plate assays confirmed UD1022’s ability to inhibit fungal growth, while *in planta* experiments showed a significant reduction in disease severity and lesion formation in treated plants. GC-MS analysis further identified key VOCs, including 2,5-dimethylbenzaldehyde and 2,4-di-tert-butylphenol, which may contribute to fungal inhibition. Gene expression analysis revealed the upregulation of key defense-related genes in the SA, JA, and ET signaling pathways, indicating a primed immune response. Collectively, these findings demonstrate UD1022 as a potent biocontrol agent with the potential to be integrated into sustainable rice blast management strategies.

## MATERIALS AND METHODS

### Fungal and bacterial cultures

The fungal strain *M. oryzae* Guy11, a model for plant-pathogen interaction studies, was cultured on CM agar (50 mL 20× nitrate salts, 1 mL trace elements, 10 g D-glucose, 2 g peptone, 1 g yeast extract, 1 g casamino acid, 1 mL vitamin solution, and distilled water to 1 L, pH adjusted to 6.5 with NaOH and solidified with 15 g agar before autoclaving at 121°C for 20 min) (56). The mycelial plugs were grown in CM and incubated at 24°C for a 12 hour light/dark cycle.

The bacterial strain *Bacillus subtilis* UD1022, a plant growth-promoting rhizobacterium, was provided by Dr. Harsh Bais (University of Delaware). Glycerol stocks were stored at −80°C and revived on LB agar (10 g/L tryptone, 5 g/L yeast extract, 5 g/L NaCl, and 18 g/L agar, pH ~7.0). Plates were incubated at 30°C overnight, and single colonies were grown in liquid LB medium under shaking at 200 rpm.

### Growth curve assay

A growth curve experiment was conducted to evaluate the bacterial growth of the *E. coli* strain DH5α in CM media. A starter culture was prepared by inoculating 5 mL of LB media with DH5α and incubating it overnight at 37°C with shaking at 200 rpm. The following morning, the OD_600_ of the starter culture was measured and adjusted to 0.1, and autoclaved 125 mL flasks containing 50 mL of either LB or CM media were each inoculated with this adjusted culture. Cultures were incubated at 25°C with shaking at 200 rpm. The OD_600_ was measured every 2–3 hours over a 12 hour period using the respective media as a blank. Optical density values were plotted against time to generate growth curves for each media condition at 25°C. Data were plotted using GraphPad Prism.

### Dual culture assay to assess direct antagonism

Overnight bacterial cultures were grown in liquid CM at 28°C with shaking at 220 rpm and normalized to an OD_600_ of 0.5 using sterile water. Fungal cores (5 mm), collected from the edges of 7-day-old fungal plates with flame-sterilized tools, were transferred to CM plates and placed 1.5 cm from the center. A 5 µL aliquot of normalized culture was added 3 cm away on the opposite side, with sterile water used as a negative control. Plates were sealed with parafilm and incubated at 25°C in the dark for 5 and 10 days. Fungal growth diameters were measured, averaged, and used to calculate the percentage of inhibition using the formula: % of inhibition = [(*D*1 − *D*2) / (*D*1)] × 100, where *D*1 = average control diameter, and *D*2 = average treatment diameter.

### Volatile-mediated assay

The volatile-mediated assay uses a stacking plate approach to evaluate the inhibitory effects of volatile organic compounds produced by bacterial isolates. CM media and bacterial cultures were prepared as described in the dual culture assay. CM plates were inoculated with 5 µL of normalized bacterial culture, spread with sterile glass beads, and incubated overnight at 28°C in the dark. Sterile water served as a negative control. Fungal cores, collected from the edges of 7-day-old fungal cultures using sterilized tools, were transferred to the center of fresh CM plates. The lid of the bacterial plate was removed, and the bacterial plate was inverted over the fungal plate to create a sealed chamber secured with parafilm. The plates were incubated for 5 days at 25°C in the dark. Fungal growth diameters were measured across the widest points, averaged, and used to calculate the percentage of inhibition as in the dual culture experiment.

### Plant material and growth conditions

*Oryza sativa* susceptible cultivars, CO39 and YT16, were gifts from Dr. Richard Wilson (University of Nebraska) and Dr. Martin Egan (University of Arkansas), respectively. Dehusked rice seeds were surface sterilized with 70% ethanol (1 min) and 5% bleach (1 hour), rinsed with sterile water, dried, and planted in the Magenta boxes. Seeds were pregerminated on half-strength Murashige and Skoog (MS) media (2.25 g/L MS medium, 30 g/L sucrose, and 0.5 g/L 2-(N-morpholino)ethanesulfonic acid (MES), pH 5.8, using potassium hydroxide, and 3 g/L gelrite). Magenta boxes were filled with 100 mL of sterilized media. Plants were grown for 10 days in a growth chamber at 25°C with a 12 hour light/dark cycle. After 10 days, plants were removed from MS media and transplanted into surface-sterilized 4-inch pots filled with autoclaved soil (2:1 Pro-Mix-HP and potting mix with perlite (Michigan Peat Baccto) and transferred to the greenhouse.

### Fungal spore germination and appressorium formation

Spores from *M. oryzae* Guy11 were harvested from 7- to 10-day-old CM agar cultures, suspended in water, and filtered through two layers of sterile Miracloth. Sterilized plastic coverslips served as hydrophobic surfaces for the conidia. A spore suspension (1 × 10⁵ spores/mL) was prepared, and bacterial suspensions of *B. subtilis* UD1022 and *E. coli* DH5α were adjusted to approximately 1.6 × 10^8^ cells/mL for treatments. Fungal spores were mixed with bacterial suspensions at a 1:1 ratio, and 20 µL was placed in a glass coverslip. Control treatments included *E. coli* DH5α and untreated spores. Treated spores (20 µL) were placed on coverslips, which were incubated in a Pyrex dish containing a wet filter to maintain humidity and incubated at 25°C. Germination percentages were assessed at 3 hpi, and appressorium formation was evaluated at 20 hpi by counting 50 conidia per technical replicate. Each experiment included three technical replicates, and the entire assay was independently repeated three times. Observations were made using a fluorescence microscope (Zeiss Axio Observer 7), and representative images were captured. Images were processed using Fiji (ImageJ distribution) (57); a faint horizontal line observed in Fig. 3 is a rendering artifact introduced during image processing. Data were analyzed and visualized using GraphPad Prism version 10.1.1 software (GraphPad Software, Inc., Boston, MA, USA).

### Gas chromatography-mass spectrometry analysis

To assess VOC production, *B. subtilis* UD1022 was cultured in 10 mL of both LB and CM media and incubated overnight at 37°C with shaking at 200 rpm. The following morning, 3.75 mL of culture from each condition was aseptically transferred into autoclaved 8 mL glass vials. Vials were sealed with rubber septa and crimped with metal caps using a vial crimping tool. VOCs were allowed to accumulate in the headspace for at least 24 hours at room temperature. A 50 µL Hamilton Gastight Sample Lock Syringe was used to collect headspace gas, with the syringe locked during insertion and opened once in the headspace. After gas collection, the syringe was sealed, and the sample was immediately injected into a Thermo Scientific TraceGOLD GC column for analysis using the Thermo Trace 1610 ISQ quadrupole GC-MS system. Between samples, the syringe was flushed with acetone three times. A blank air sample was also analyzed as a control. The GC program included a 2 min hold at 70°C, followed by a temperature ramp to 300°C at 10°C/min, with a total run time of 27 min. Mass spectra were identified by comparing the results to reference spectra in the NIST Mass Spectral Library. Three independent experiments were performed for this assay.

### Infection assays

Bacterial cultures were prepared by growing samples in LB broth at 30°C and 220 rpm overnight. Cultures were normalized to approximately 1.6 × 10⁸ cells/mL, washed twice with sterile water, and resuspended in 10 mL of sterile water. Ten-day-old plants were inoculated by submerging roots in bacterial suspensions for 20 min before being transplanted into sterile soil pots. At 3 weeks, the plants were reinoculated with 10 mL of bacterial culture added to the soil surrounding each plant.

After 24 hours, the plants were sprayed with an *M. oryzae* spore suspension (1 × 10⁵ spores/mL in 0.2% gelatin). Inoculation and infection experiments were conducted in a growth chamber. To maintain high humidity, the plants were covered with plastic bags for 48 hours and incubated in a growth chamber at 25°C with a 12 hour light/dark cycle. Bags were partially opened after 2 days to reduce humidity. After 5 days, infected leaves were collected for imaging and further gene expression analysis. Disease symptoms were quantified by measuring the percentage of disease-covered areas using Fiji ImageJ software (57).

### Gene expression analysis

Following inoculation with bacterial isolates and fungal infections, leaves were collected for RNA extraction using a plant RNA isolation kit (RNeasy Plant Mini Kit, Qiagen), following the manufacturer’s protocol. The quality and concentration of the RNA were assessed using a spectrophotometer. First-strand complementary DNA (cDNA) was synthesized from 1 µg of total RNA using a reverse transcription kit with oligo-dT primers (QuantaBio). Quantitative PCR was performed using a SYBR Green-based detection system in a thermal cycler (BioRad). Reactions were conducted in triplicate for each sample, with a total volume of 10 µL per reaction, containing cDNA, primers specific to the target gene (Table S1), and SYBR Green master mix (QuantaBio). Amplification conditions included an initial denaturation step, followed by 40 cycles of denaturation, annealing, and extension at optimized temperatures. Expression levels of the target genes were normalized against the housekeeping gene *GAPDH*. The comparative CT method (*ΔΔCT*) was used to calculate relative gene expression levels compared to an uninoculated control. Data from three biological replicates were included for statistical analysis. Graphs and statistical analyses were performed using GraphPad Prism version 10.1.1 (GraphPad Software, Inc.). Results were visualized as fold changes in gene expression, with statistically significant differences determined using appropriate tests (*P* < 0.05). Error bars represent the standard deviation of biological replicates.

### Statistical analysis

Statistical analysis was performed using one-way analysis of variance for comparisons across multiple conditions and Student’s *t*-test. All assays included at least three biological replicates. Results were considered statistically significant when *P* < 0.05. Exact *P* values are annotated in the figures. Data were analyzed and visualized using GraphPad Prism 10.1.1 software (GraphPad Software, Inc.).

## SUPPLEMENTAL MATERIAL

Supplemental material spectrum.00797-25-s0001.docx Fig. S1 and S2; Table S1.

ASM does not own the copyrights to Supplemental Material that may be linked to, or accessed through, an article. The authors have granted ASM a non-exclusive, world-wide license to publish the Supplemental Material files. Please contact the corresponding author directly for reuse.
