# Supplementary material for: Bacillus subtilis strain UD1022 as a biocontrol agent against Magnaporthe oryzae, the rice blast pathogen
Source: Microbiol Spectr. 2025 Sep 22;13(11):e00797-25. doi: 10.1128/spectrum.00797-25 (PMC12584654; doi:10.1128/spectrum.00797-25)
Supplement: Supplemental material — Fig. S1 and S2; Table S1. [file spectrum.00797-25-s0001.docx]

**Supplemental Material**

**
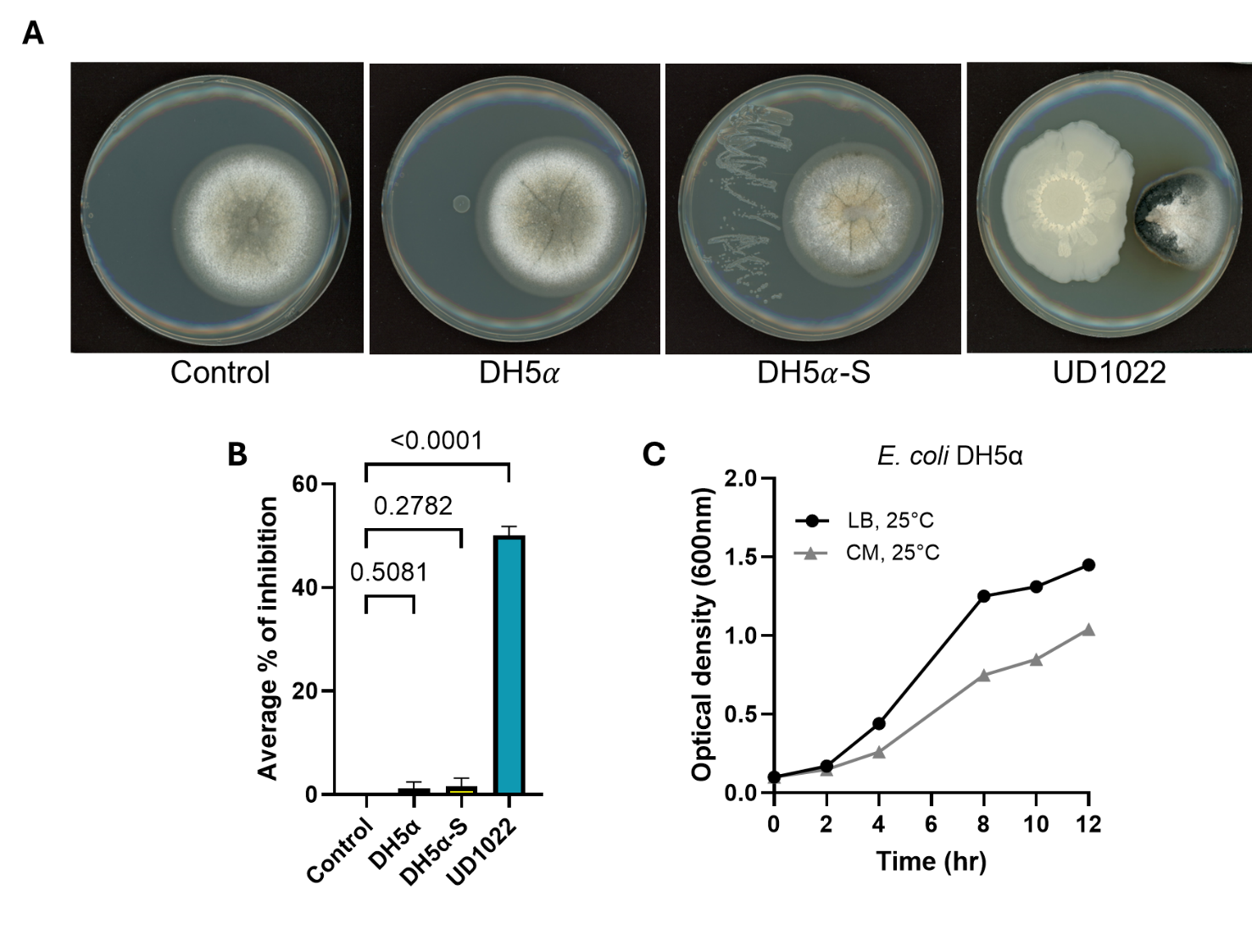
Figure S1**

**Figure S1. Antagonistic activity of *B. subtilis* UD1022 against *M. oryzae.*** **A.** Dual culture assay on complete medium (CM) plates, incubated at 25 °C for 10 days. Images and fungal growth measurements were taken at 10 days post-incubation (dpi). **B.** Quantification of fungal growth inhibition (%) in the presence of UD1022 and control treatments: *E. coli* DH5α, manually streaked DH5α (DH5α-S), and water. Bars represent the mean ± SD from three independent biological replicates (*n* = 3). Statistical analysis was performed using one-way ANOVA followed by Dunnett’s multiple comparison test. Exact *p*-values are indicated on the graph. **C.** Growth curve of *E. coli* DH5α in LB and CM liquid media at 25 °C. Optical density (OD_600_) measurements were recorded every 2–3 hours over a 12-hour incubation period.

**Figure 2S**

**
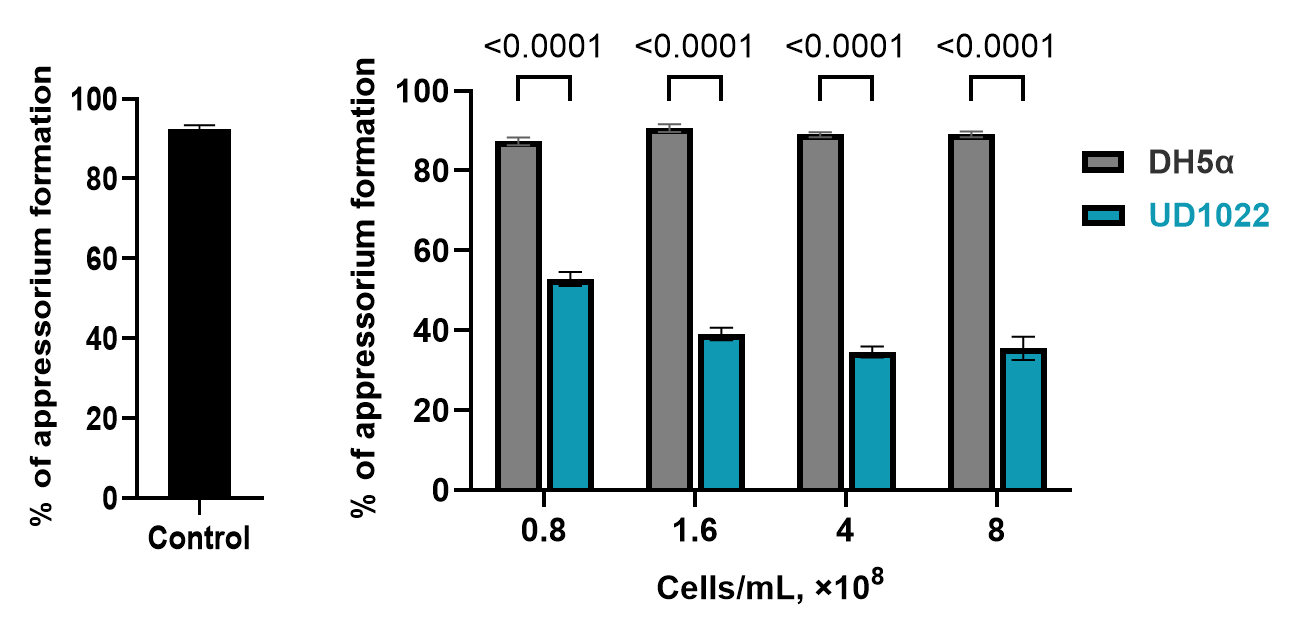
**

**Figure S2**. **UD1022 inhibits *M. oryzae* appressorium formation at different concentrations.** Effect of *B. subtilis* UD1022 on *M. oryzae* appressorium formation through different bacterial concentrations. Fungal spores (1 x 10^5^ spores/mL) were mixed with bacterial suspensions of varying concentrations and incubated in a 20 µL drop on an appressorium-inductive surface (hydrophobic coverslip). Appressorium formation was evaluated at 20 hpi. Bar graphs represent the percentage of *M. oryzae* appressorium formation under control conditions (black) and in the presence of UD1022 (blue) and *E. coli* DH5α. Error bars indicate standard deviation. Statistical significance was determined using one-way ANOVA (p < 0.05).

**Supplementary Table 1**. List of PCR primers used for qRT-PCR.

| **Names** | **Primers Sequence (5’-3’)** | **Source** |
| --- | --- | --- |
| PAD4 | Forward: GCACAAGTTTGAGCGCCATT  Reverse: ACTCCTTCACCCACAGGGAGTA | (1) |
| EDS1 | Forward: TTGAATTTTGTCGTGCCAGTAGA  Reverse: GGCAGATGCAAGCGGAGTAA | (2) |
| WRKY45 | Forward: CGGGTAAAACGATCGAAAGA  Reverse: TTTCGAAAGCGGAAGAACAG | (3) |
| EIL1 | Forward: ACAATGCCACGATCATGGAG  Reverse: TCAGTAGTACCAATTCGAGC | (1) |
| EIN2 | Forward: CAAGGAACCAGTGACAACCA  Reverse: GCAGTCGTCTCCGCAGTTAG | (4) |
| PR10b | Forward: AGGTGTTGGAGGTTAAGAGC  Reverse: TCGACGATCATCTTCTCCTG | (5) |
| ERF1 | Forward: CATATCACCTTGACGCCCCA  Reverse: ACCCTCACAAACTCACTCGG | (6) |
| WRKY30 | Forward: ACTTCTTGAGTCGCCGGTTT  Reverse: GCTTCTGGGATGCTCACTGT | (6) |
| WRKY77 | Forward: CTGTGTCCAGCTACCTCTCC  Reverse: TGAAGAGAGCGATCACCTC | (6) |
| JAR1 | Forward: TCTCCCCAGCCTTAACCGTA  Reverse: CTAAACGCGACGACAAACCC | (6) |
| GAPDH | Forward: AAGCCAGCATCCTATGATCAGATT  Reverse: CGTAACCCAGAATACCCTTGAGTTT | (7) |

**REFERENCES**

1. Ke Y, Liu H, Li X, Xiao J, Wang S. 2014. Rice Os4 functions differently from *Arabidopsis* At4 in host-pathogen interactions. The Plant Journal 78:619-631. <https://doi.org/10.1111/tpj.12500>.

2. Patel A, Sahu KP, Mehta S, Balamurugan A, Kumar M, Sheoran N, Kumar S, Krishnappa C, Ashajyothi M, Kundu A, Goyal T, Narayanasamy P, Kumar A. 2022. Rice leaf endophytic *Microbacterium testaceum*: Antifungal actinobacterium confers immunocompetence against rice blast disease. Frontiers in Microbiology 13. [https://doi.org/10.3389/fmicb.2022.1035602](https://doi.org/).

3. Akagi A, Fukushima S, Okada K, Jiang C-J, Yoshida R, Nakayama A, Shimono M, Sugano S, Yamane H, Takatsuji H. 2014. WRKY45-dependent priming of diterpenoid phytoalexin biosynthesis in rice and the role of cytokinin in triggering the reaction. Plant Molecular Biology 86:171-183. [https://doi.org/10.1007/s11103-014-0221-x](https://doi.org/).

4. Duan C, Yu J, Bai J, Zhu Z, Wang X. 2014. Induced defense responses in rice plants against small brown planthopper infestation. The Crop Journal 2:55-62. <https://doi.org/10.1016/j.cj.2013.12.001>.

5. Valette M, Rey M, Doré J, Gerin F, Wisniewski-Dyé F. 2020. Identification of a small set of genes commonly regulated in rice roots in response to beneficial rhizobacteria. Physiology and Molecular Biology of Plants 26:2537-2551. [https://doi.org/10.1007/s12298-020-00911-1](https://doi.org/).

6. Spence C, Alff E, Johnson C, Ramos C, Donofrio N, Sundaresan V, Bais H. 2014. Natural rice rhizospheric microbes suppress rice blast infections. BMC Plant Biol 14:130. [https://doi.org/10.1186/1471-2229-14-130](https://doi.org/).

7. Chen X, Laborda P, Dong Y, Liu F. 2020. Evaluation of suitable reference genes for normalization of quantitative real-time PCR analysis in rice plants under *Xanthomonas oryzae* pv. oryzae-infection and melatonin supplementation. Food Production, Processing and Nutrition 2:21. [https://doi.org/10.186/s43014-020-00035-9](https://doi.org/).
